# Development and validation of a case-finding algorithm for the identification of non-small cell lung cancers in a region-wide Italian pathology registry

**DOI:** 10.1371/journal.pone.0269232

**Published:** 2022-06-08

**Authors:** Andrea Spini, Pietro Rosellini, Cristiana Bellan, Folco Furiesi, Silvano Giorgi, Sandra Donnini, Rosa Gini, Marina Ziche, Francesco Salvo, Giuseppe Roberto

**Affiliations:** 1 INSERM, BPH, U1219, Team Pharmacoepidemiology, University of Bordeaux, Bordeaux, France; 2 Department of Medical Science, Surgery and Neuroscience, University of Siena, Siena, Italy; 3 Pole de Santé Publique, Service de Pharmacologie Médicale, Centre de Pharmacovigilance de Bordeaux, CHU de Bordeaux, Bordeaux, France; 4 CIC1401, CIC Bordeaux, Bordeaux, France; 5 Department of Medical biotechnology, University of Siena, Siena, Italy; 6 Azienda Ospedaliera Universitaria Senese, Siena, Italy; 7 Department of Life sciences, University of Siena, Siena, Italy; 8 Osservatorio di Epidemiologia, Agenzia regionale di sanità della Toscana, Florence, Italy; University of Oxford, UNITED KINGDOM

## Abstract

**Purpose:**

To develop and validate a case-finding algorithm for the identification of Non-Small Cell Lung Cancer (NSCLC) cases in a region-wide Italian pathology registry (PR).

**Materials and methods:**

Data collected between 2009 and 2017 in the PR and the Pharmacy Database of the University Hospital of Siena and the PR of Tuscany region were used. A NSCLC-identification algorithm based on free-text keywords and SNOMED morphology and topography codes was designed and tested on data from Siena: indication for drug use (i.e. NSCLC) was the reference standard for sensitivity (SE); positive predictive value (PPV) was estimated through manual review. Algorithm modifications were then tested to improve algorithm performance: PPV was calculated against validated dataset from PR of Siena; a range of SE [min-max] was estimated in PR of Tuscany using analytical formulae that assumed NSCLC incidence equal either to 80% or 90% of overall lung cancer incidence recorded in Tuscany. The algorithm modification with the best performance was chosen as the final version of the algorithm. A random sample of 200 cases was extracted from the PR of Tuscany for manual review.

**Results:**

The first version of the algorithm showed a PPV of 74.7% and SE of 79% in PR of Siena. The final version of the algorithm had a SE in PR of Tuscany that grew with calendar time (2009 = [24.7%-28%]; 2017 = [57.9%-65.1%]) and a PPV of 93%.

**Conclusions:**

The final NSCLC-finding algorithm showed with very high PPV. SE was in line with the expected contribution of PR to overall cases captured in the regional Cancer Registry, with a trend of increase over calendar time. Given the promising algorithm validity and the wide use of SNOMED terminology in electronic pathology records, the proposed algorithm is expected to be easily adapted to other electronic databases for (pharmaco)epidemiology purposes.

## Introduction

Lung cancer is the most commonly diagnosed cancer worldwide (2.09 million cases in 2018) with approximatively 42.500 new diagnoses in Italy in 2019 [1]. Non-small-cell lung cancer (NSCLC) represents about 85% of all cases of lung cancer and can be distinguished in three main histotypes: squamous, big cells, and adenocarcinoma [2]. ln the past fifteen years, a number of novel anticancer medications, such as target- and immunotherapies, have been authorized for the pharmacological treatment of advanced stage NSCLC [3]. Nevertheless, knowledge on the efficacy and safety of such anticancer drugs mostly relies on evidence from clinical trials which are usually based on relatively small samples of selected patient populations [4, 5].

Large databases of routinely collected electronic healthcare data can be used to generate evidence on the epidemiology of diseases as well as on the real-world utilization of medicines in population that were not included in clinical trials [6–8]. However, validity of variables retrieved from healthcare data is imperfect, and its measurement is necessary to assess reliability of such evidence [9].

The aim of the study was to develop and to validate a case-finding algorithm for the identification of patients with NSCLC in a region-wide pathology registry in Italy.

## Material and methods

### Data sources

For the purposes of this study, three data sources were used: 1) the pathology registry of the University Hospital of Siena, which is a city in Tuscany that hosts the University Hospital “Le Scotte”, one of the major hospitals of the region, 2) the Hospital Pharmacy database of Siena and 3) the region-wide administrative healthcare database of Tuscany.

- Pathology Registry of the University Hospital of Siena

The pathology registry of Siena collects records of histological and cytological examinations of patients attending the University Hospital of Siena. It contains patient demographics and pathological diagnoses, which are both recorded using the “Systematized Nomenclature Of Medicine” (SNOMED) morphology and topography codes, and free-text description of macroscopical and microscopical findings, as well as other clinical information. Biomolecular characterization (i.e., EGFR positive mutations) can be also recorded with SNOMED codes.

- The Hospital Pharmacy database of Siena

The Hospital Pharmacy database of Siena (HPS) collects information concerning intravenous drugs prepared by the hospital pharmacy and administered to both inpatients and outpatients treated at the Siena University Hospital. The database contains demographic data of treated patients as well as information on the administered drug, including indication of use.

- Regional administrative healthcare database of Tuscany

The administrative healthcare database of Tuscany collects information on all healthcare services dispensed to Tuscan inhabitants (around 3.7 million subjects) and reimbursed by the National Healthcare Service. This database includes multiple registries that can be linked with each other through a pseudo-anonymized regional person identifier code. In this study, two registries were used: the inhabitant registry, which records demographic information, including vital status of all inhabitants entitled to public healthcare assistance, and the regional pathology registry. The latter covers the whole Tuscan population and contains all variables available in the pathology registry of Siena, except for molecular characterization.

### Training dataset

All the records collected in the pathology registry of Siena between 01/01/2009 and 31/08/2017 were extracted and used as training dataset. Moreover, patients with at least one record concerning the administration of a drug indicated for NSCLC were also extracted from HPS. The competent authority assigned the regional pseudo-anonymized ID to each patient identified in HPS and the pathology registry of Siena, respectively. Using the regional pseudo-anonymized ID, patient-level information was linked to the inhabitant registry of Tuscany to include only Tuscan residents.

### Design of a case-finding algorithm for NSCLC identification

The first version of the case-finding algorithm for the identification of NSCLC cases was designed by a multi-expertise work group including clinicians from the Pathology Department of the Siena University Hospital, epidemiologists, and information technology experts. The population of cases the algorithm was intended to identify corresponded to the intersection of cases identified by a morphological (Morpho_1) and a topographical component (Topog_1) (Table 1). Morpho_1 was developed to identify histologies that fall within the pathological definition of NSCLC, while Topog_1 aimed at identifying the lung site. For both components, topography, and morphology SNOMED codes together with keywords from the free text diagnosis field were used.

**Table 1:** Components of the first version of the algorithm used for the identification of NSCLC cases in the pathology registry of Siena*.

Acronym of algorithm’s component	Description	Tumoral characteristic	Terminology	Codes/free text words	Criteria
**Morpho_1**	NSCLC codes and free text	Morphology	SNOMED and free text	M-81403 OR M-80703 OR M-82463 OR M-85603 OR M-83233 OR-85503 OR M-84303 OR M-82503 OR M-80033 OR M-80123 OR M-80463 OR (M-8* AND (Text in diagnosis field:“*non microcit*" OR "*adenocarcin*" "*squamocell*" OR "*grandi cell*”))	Inclusion
**Topog_1**	Chest topography	Topography	SNOMED and free text	T-2* OR Text in diagnosis filed: “POLM*” OR “BRONCH*”	Inclusion

* First version of the algorithm corresponded to the intersection of the two components reported in the table, i.e. Morpho_1 AND Topog_1.

In developing the first version of the algorithm, we applied an exploratory strategy, meant to target an appropriate balance between decreasing false negatives (improve sensitivity—SE) and increasing true positives (improve positive predictive value—PPV). To do so, we calculated an indicator of sensitivity: the number of subjects extracted by the algorithm as a share of those treated for NSCLC in HPS. This provided us with an indication of the sensitivity of the algorithm. Therefore, we selected those keywords that substantially increased this indicator without dramatically increasing the total number of cases extracted.

### Validation of the first version of the NSCLC case-finding algorithm against the training dataset

While an indicator of algorithm sensitivity was obtained against patients with an administration of a drug indicated for NSCLC in HPS, PPV was calculated based on the results from a manual review of records of all cases of NSCLC identified in the pathology registry of Siena (*reference standard*). Information used for the manual review was extracted from the free-text fields (pathological diagnosis, macroscopy, microscopy, clinical information and sent material) of the first record identified for each patient during the study period. The information was extracted manually and independently by two researchers (AS and PR) which worked blinded to each other. A senior pathologist (CB) from Siena University Hospital resolved disagreements between the two assessors. Data were extracted in a standard extraction form, encompassing four items: 1) Malignancy: Malignant tumor, Yes/No/Maybe; 2) Topography: Lung, Yes/No/Maybe; 3) Morphology: NSCLC, Yes/No/Maybe; and 4) Origin of the tumor: primary, Yes/No/Maybe. Those cases that received “*yes”* in all the four validation items, or those who received “*yes”* in the first three validation items and “*maybe”* with respect to the primary origin of the tumor were considered as confirmed NSCLC cases. Cohen’s Kappa for concordance between assessors was also calculated.

### Refinement of the first version of the NSCLC case-finding algorithm: Validation of different algorithm modifications

Based on the experience from the manual review, we tested the impact of several algorithm modifications. Namely, a set of additional algorithm *components* were used as exclusion criteria with respect to the population of cases identified by the first version of the algorithm. The validity of the tested algorithms modifications was then estimated as follow:

- PPV: the validated dataset extracted from pathology registry of Siena (see above) was used as the reference standard.
- SE: analytical formulae based on algebraic interrelation among PPV, SE, observed incidence (P), and true incidence (∏) were used [10, 11]. P was obtained from direct application of the relevant algorithm variant in the pathology registry of Tuscany. ∏ was obtained from the most recent estimate of overall lung cancer incidence in the Region, i.e. 2014, reported from the Cancer Registry of Tuscany [12]: although slight regional variations cannot be excluded, NSCLC is widely accepted as representing about 85% of all lung cancer cases [1, 13], thus two extreme values of ∏, corresponding to 80% and 90%, respectively, of the overall lung cancers in order to obtain a range [min-max] of SE values were considered. We also assumed that ∏ did not change during study period.

The combination of algorithm components with the best performance in terms of SE and PPV was then selected as final version of the algorithm.

### Application of final version of the NSCLC case-finding algorithm to the pathology registry of Tuscany

The PPV of the final version of the algorithm in the general Tuscan population was estimated in the pathology registry of Tuscany. A random sample of 200 cases retrieved from 2009 to 2019 was extracted and manually validated by one researcher (AS) who consulted two other experts (PR and CB) for resolving any doubt on the assessment.

### Ethical statement

The study was approved by the local ethic committee (Ethical committee Area Vasta Sud Est, Siena, Italy; Project: ARS-AOUS-2017) and waived the requirement for informed patient consent given the retrospective nature of the study, and de-identified nature of the data.

## Results

### Application of the first version of the algorithm and validation against the training dataset

SNOMED codes and free-text keywords selected for the components Morpho_1 and Topog_1 of the first version of the algorithm were reported in Table 1 (more details on SNOMED codes and strings describing each code were reported in S1 Table; also translation of Italian keywords to English were reported in S2 Table). The algorithm allowed to retrieve 2309 cases from the pathology registry of Siena during the period 01/01/2009-31/08/2017, of which 2003 were linkable to the inhabitant registry of the regional administrative healthcare database of Tuscany and, thus, were considered for manual chart validation.

As for PPV, out of 2003 cases labeled as NSCLC cases, 1496 were classified as confirmed cases, leading to an estimate of 74.7% for PPV (see S3 Table for results). Cohen’s k measure of concordance between reviewers was as follows: 1) k = 0.1350 for the malignancy item (slight agreement); 2) k = 0.7617 for the lung item; (substantial agreement); 3) k = 0.4307 for the morphology item (moderate agreement); 4) k = 0.3128 for the origin item (fair agreement).

Patients receiving a drug treatment with a recorded NSCLC indication in HPS were 469: among those, 373 were identified by the first version of the algorithm, leading to a SE indicator of 79.5%.

### Refinement of the first version of the NSCLC case-finding algorithm: Validation of different algorithm modifications

Manual validation identified SNOMED codes and free-text keywords that were used to design additional algorithm components as exclusion criteria for the improvement of the first version of the algorithm (Table 2). Two algorithm modifications were obtained based on different logical combination of additional algorithm components (Table 3).

**Table 2:** Additional algorithm components used for algorithm modifications.

Acronym of algorithm’s component	Description	Tumoral characteristic	Terminology	Codes/free text words	Criterion
**Morpho_K_neuroend**	Neuroendocrin carcinoma	Morphology	SNOMED	M-82463	Exclusion
**Topog_Upper_airways**	Upper airways	Topography	SNOMED	T-21* OR T-22* OR T-23* OR T-24* OR T-25*	Exclusion
**Topog_Resp_Not_specified**	Respiratory system not specified	Topography	SNOMED	T-20*	Exclusion

**Table 3:** First version of the algorithm and algorithm modifications.

**First version of the algorithm**
Morfo_1 AND Topog_1
**First modification**
(Morfo_1 AND NOT Morfo_K_neuroend) AND ((Topog_1 AND NOT (**Topog_Upper_airways** OR **Topog_Resp_Not_specified**))
**Second modification** *
(Morfo_1 AND NOT Morfo_K_neuroend) AND (Topog_1 AND NOT **Topog_Upper_airways**)

*Chosen as final version of the algorithm

**Fig 1:**
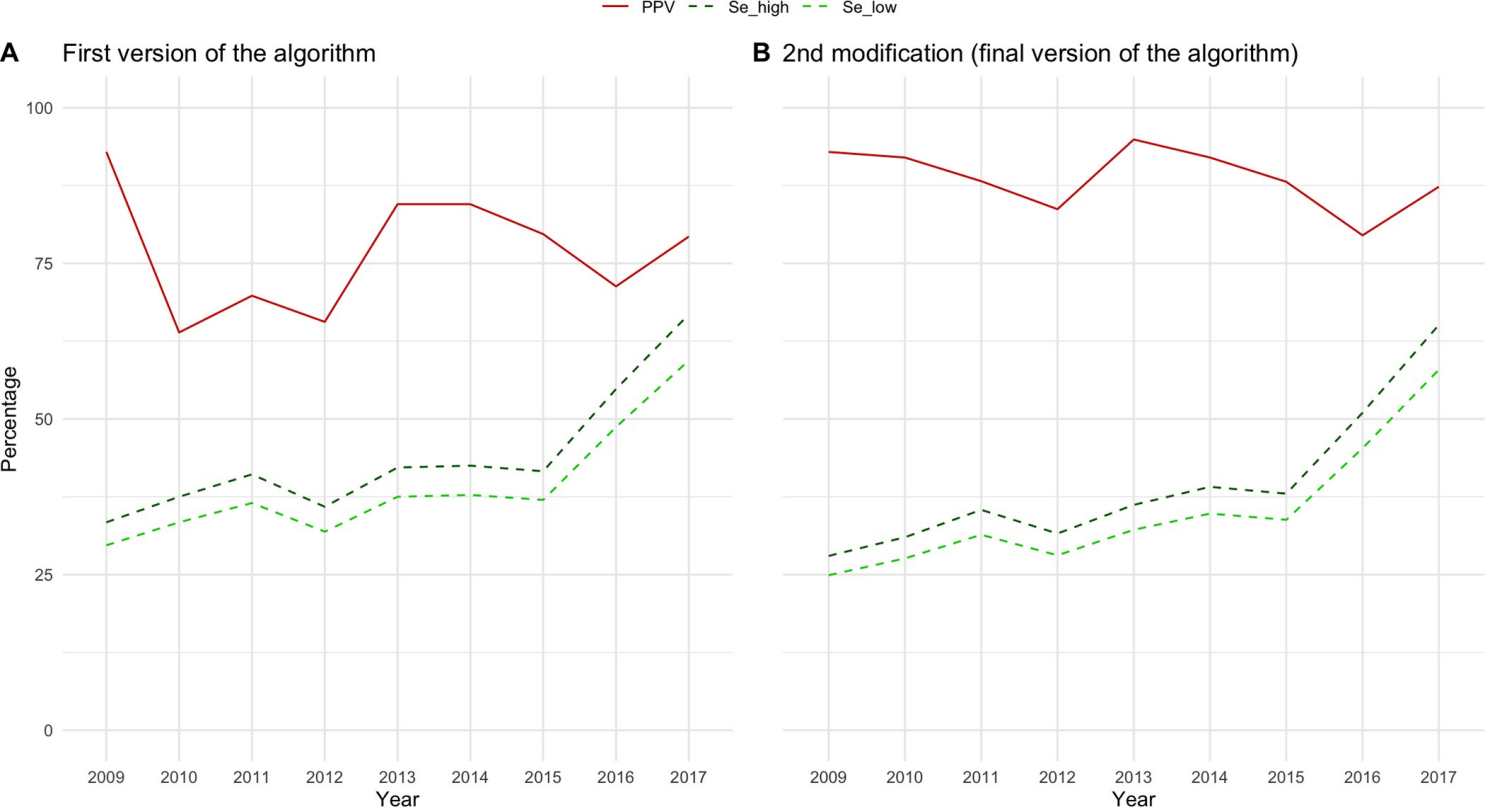
Positive predictive value (PPV) and sensitivity (SE) of first version and final version of the algorithm. The figure shows the validity measures of the first and the final version of the algorithm. PPV was represented in red and SE in green (dark green–higher value of SE; light green–lower value of SE).

The two algorithm modifications showed similar results in terms of PPV and SE. The algorithm modification with the best performance showed a PPV of 87.9% and an increasing sensitivity over the years (Fig 1): in 2009 SE range was [min = 24.9% (IC95%: 23.1–26.7)-max = 28.0% (IC95%: 26.3–29.7)] while in 2017 SE range was [min = 57.9%(IC95%: 55.9–59.9)–max = 65.1%(IC95%: 63.3–66.9)] (in S1 Fig of the results from both algorithm modifications are reported). This algorithm modification was then chosen as final version of the NSCLC case-finding algorithm for the regional pathology registry of Tuscany.

### Performance of final version of the algorithm in regional pathology registry of Tuscany

The algorithm was applied to the records collected in the pathology registry of Tuscany between 2009 and 2019. Overall, 15,169 cases were identified. A random sample of 200 cases was validated manually (see S3 Table of the full report of validation assessment). The number of confirmed NSCLC cases was 186/200, corresponding to a PPV of 93%.

## Discussion

In this paper we reported our experience concerning the development and validation of a case-finding algorithm for the identification of NSCLC cases in a region-wide Italian pathology registry. The algorithm was found to have an high PPV and an acceptable SE which estimates appeared to increase over the study period and could be considered in line with the share of regional NSCLC cases that are likely to be captured by the pathology registry [14]. Notably, two possibly concurrent reasons can explain the observed trend of increase of the estimated SE. First, the regional pathology registry of Tuscany was established in 2006 so that completeness of data recording was expected to increase over the years. Second, the establishment of clinically and scientifically accredited regional centers during the last decade might have contributed to attract an increasing number of Tuscan inhabitants towards Tuscan hospitals, rather than to other out-of-the-region facilities. Thus, over the study period, the regional pathology registry of Tuscany have reasonably accounted for an increasing share of the total NSCLC cases captured by the regional Cancer Registry, which is also fed by hospital discharge records, including those concerning admissions of Tuscan patients to extra-regional facilities, and death certificates [14].

The process adopted in this study for developing the final version of the NSCLC-finding algorithm was stepwise. Manual ascertainment of the 2003 cases extracted through the application of the first version of the algorithm to the training dataset allowed to identify two additional exclusion criteria, concerning neuroendocrine morphology and upper airways topography, respectively. Adding the latter two components to the first version of the algorithm allowed to increase PPV without compromising SE.

Given the promising evidence on validity reported in this work, the final version of the NSCLC-finding algorithm designed and tested in this study can be considered suitable for epidemiology and pharmacoepidemiology studies. In fact, the proposed case-finding algorithm was recently used to identify a cohort of NSCLC patients in a recently published pharmacoepidemiological study [15]. Notably, the characteristics of the study cohort were in line with those expected from the disease epidemiology [1, 2]. Pathology registries can therefore provide a valid complement to other data sources that are widely used for research purposes, such as hospital discharge records, where information on histology is often lacking [16]. Notably, information on pathological staging (TNM) and histology can be also found in the pathology registry, however assessing the completeness and validity of such variables in the pathology registry of Tuscany was out of the scope of this work.

This study has several strengths. First, to the best of our knowledge this is the first validation study concerning the application of a NSCLC case-finding algorithm in an Italian pathology registry. Since the algorithm developed in this study was based on SNOMED codes and free-text keywords, this work will serve as the starting point for local NSCLC case-finding algorithm design and application. In fact, SNOMED terminology is widely used in electronic pathology reports [17] so that the proposed algorithm is expected to be easily adapted to other electronic databases. Second, two manual case validations were performed in this study. In particular, the double-blinded manual validation of the 2003 cases identified in the training dataset (i.e., the pathology registry of Siena) allowed gaining insight into the specific codes and keywords used in the pathology reports which were finally adopted for algorithm refinement. Future studies that will intend to identify NSCLC cases in a pathology registry will be able to leverage the present work by adapting the algorithm proposed here to the specific characteristics of the local data (e.g., translating in any other language of interest the keywords used for free text search–see S2 Table). Third, an innovative methodology for estimating missing validity indices was used to obtain estimate of algorithm sensitivity [10, 11]. With this respect, evidence from a recent literature review demonstrates that validation studies often report PPV only, while sensitivity is rarely assessed (i.e., measurement of sensitivity may not be feasible when the outcome is rare and sometimes is difficult to obtain) [18].

This study has also limitation that deserve to be acknowledged. The databases of the pathology registry and the HPS respectively collect pathological examinations and administrations of intravenous drugs to both inpatients and outpatients attending the Hospital of Siena. Given the nature of these data sources, we do expect high validity and completeness of the information collected, though objective estimates of are not available. For this reason, we referred to these databases as “reference standard” rather than “gold standard”, as suggested by Ehrenstein et al. 2021 [19]. Another limitation of this study concerns the estimates of the algorithms’ SE. The indicator that we used in the design phase implied assuming that the SE was equal irrespective to treatment status of patients with NSCLC and it was only functional to guide the algorithm development. However, the estimates of SE obtained when the same version of the algorithm was tested against the Regional Cancer Registry were comparable (Fig 1). For the final version of the algorithm, the adopted reference standard was the most recent estimate of the overall lung cancer incidence from the regional Cancer Registry of Tuscany (i.e., 2014), which we assumed that remained stable during the whole study period. Considering the evidence of a possible trend of slight decrease of the overall lung cancer incidence during the same period [20], the latter assumption might have led to an under-estimation of SE of the final version of the proposed NSCLC-finding algorithm from 2014 onward (in 2014 the SE max was 39% and min 35%). Notably, incidence of overall lung cancer cases was only available so we used two extreme values of the expected share of NSCLC cases (80%-90%) which allowed to obtain a range of SE estimates [2], within which the true SE was likely to actually fall. Moreover, the regional pathology registry only captures patients diagnosed in Tuscany while the Cancer registry of Tuscany also captures Tuscan inhabitants diagnosed in extra-regional facilities. This partly explains the sensitivity of the final version of the algorithm applied to the regional pathology registry when compared to the estimates from the regional cancer registry. Notably, the opposite phenomenon is not expected to happen: persons who are residents outside the region and diagnosed in a Tuscan healthcare facility cannot be captured neither in the regional pathology registry, nor in the regional cancer registry since both sources are restricted to patients who are registered to the inhabitant registry of Tuscany. Another study limitation concerns the classification of confirmed cases based on the results of the manual review. PPV value was calculated considering as confirmed NSCLC cases also those for which the primary origin of the tumor could not be identified based on all the available information recorded. This choice was made because pathology registries do not always contain information on the tumor’s origin (see S3 Table). However, to reduce possible misclassification of secondary NSCLC, the first available record in the pathology registry was considered for each patient. Nevertheless, for future applications of the present algorithm, other sources of data should be used whenever linkable to pathology registry in order to refine the selection of primary NSCLC cases (e.g., hospital discharge registries).

In conclusion, with this study we designed and validated a case-finding algorithm for the identification of NSCLC cases in a region-wide pathology registry. The algorithm was found to have a high PPV (i.e., 93%) and an acceptable SE which estimates appeared to increase over the study period and could be considered in line with the share of regional NSCLC cases that are expected to be captured by the pathology registry. Given its promising validity the proposed NSCLC-finding algorithm can be considered suitable for future epidemiology and pharmacoepidemiology studies. Given the wide use of SNOMED terminology in electronic pathology reports, the proposed algorithm is expected to be easily adapted to other electronic databases.

## Supporting information

S1 Fig Positive predictive value (PPV) and sensitivity (SE) of first version of the algorithm and both algorithm modifications. The figure shows the validity measures of the first and both algorithm modifications. PPV was represented in red and SE in green (dark green–higher value of SE; light green–lower value of SE).
(TIF)

S1 Table SNOMED morphology codes description. The table shows for each specific code the respective description.
(DOCX)

S2 Table Keywords translation in English. The table shows the translation of each Italian keywords to English.
(DOCX)

S3 Table Manual validation results of first version and final version of the algorithm. The table shows the results of the manual validation (malignancy; topography; morphology and origin) of the first and the final version of the algorithm.
(DOCX)
