# Supplementary figures and images for: Development and validation of a case-finding algorithm for the identification of non-small cell lung cancers in a region-wide Italian pathology registry
Source: PLoS One. 2022 Jun 8;17(6):e0269232. doi: 10.1371/journal.pone.0269232 (PMC9176782; doi:10.1371/journal.pone.0269232)

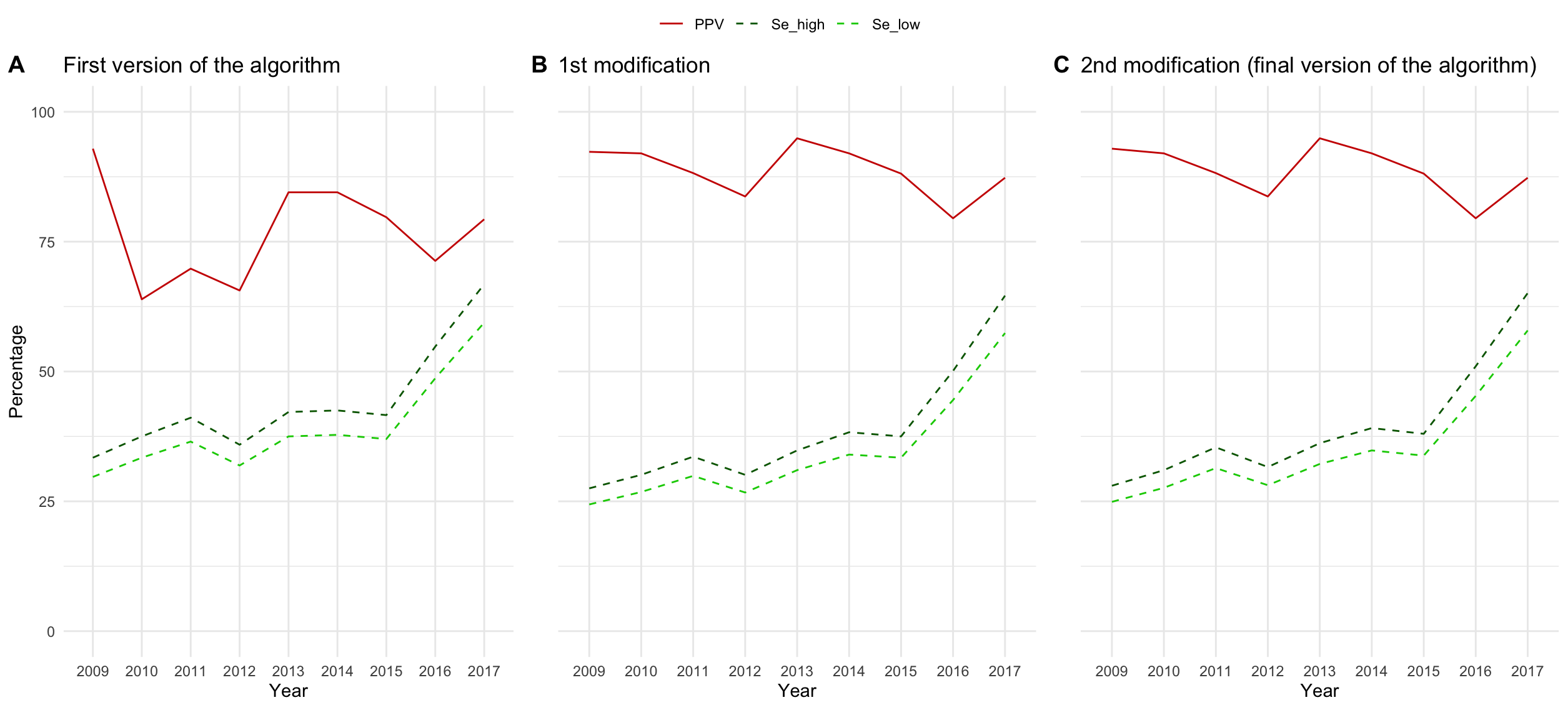

Supplement: S1 Fig — The figure shows the validity measures of the first and both algorithm modifications. PPV was represented in red and SE in green (dark green–higher value of SE; light green–lower value of SE). (TIF) [file pone.0269232.s001.tif]
