# Supplementary material for: Development and validation of a case-finding algorithm for the identification of non-small cell lung cancers in a region-wide Italian pathology registry
Source: PLoS One. 2022 Jun 8;17(6):e0269232. doi: 10.1371/journal.pone.0269232 (PMC9176782; doi:10.1371/journal.pone.0269232)
Supplement: S1 Table — The table shows for each specific code the respective description. (DOCX) [file pone.0269232.s002.docx]

**S1 Table.** **SNOMED codes description.**

| **Morphologic codes** | **Description** |
| --- | --- |
| M-81403 | Adenocarcinoma, NOS |
| M-80703 | Squamous cell carcinoma, NOS |
| M-82463 | Neuroendocrine carcinoma |
| M-85603 | Adenosquamous carcinoma |
| M-83233 | Mixed cell adenocarcinoma |
| M-85503 | Acinar cell adenocarcinoma |
| M-84303 | Mucoepidermoid carcinoma |
| M-82503 | Bronchiolo-alveolar adenocarcinoma |
| M-80033 | Malignant tumor, giant cell type |
| M-80123 | Large cell carcinoma, NOS |
| M-80463 | Non-small cell lung cancer, NOS |
| M-8* | Codes that represent reportable neoplasms |

NOS: Not otherwise specified
