# Supplementary material for: Development and validation of a case-finding algorithm for the identification of non-small cell lung cancers in a region-wide Italian pathology registry
Source: PLoS One. 2022 Jun 8;17(6):e0269232. doi: 10.1371/journal.pone.0269232 (PMC9176782; doi:10.1371/journal.pone.0269232)
Supplement: S2 Table — The table shows the translation of each Italian keywords to English. (DOCX) [file pone.0269232.s003.docx]

**S2 Table.** **Keywords translation in English.**

| **Algorithm keywords** | **Translation** |
| --- | --- |
| Non microcit | Non-small cell |
| Adenocarcin | Adenocarcinoma |
| Squamocell | Squamous/epidermoid |
| Grandi cell | Large cell |
| Polm | Lung |
| Bronch | Bronchus/Bronchioles |
