# Supplementary material for: Development and validation of a case-finding algorithm for the identification of non-small cell lung cancers in a region-wide Italian pathology registry
Source: PLoS One. 2022 Jun 8;17(6):e0269232. doi: 10.1371/journal.pone.0269232 (PMC9176782; doi:10.1371/journal.pone.0269232)
Supplement: S3 Table — The table shows the results of the manual validation (malignancy; topography; morphology and origin) of the first and the final version of the algorithm. (DOCX) [file pone.0269232.s004.docx]

**S3 Table. Manual validation results of first version and final version of the algorithm.**

| **Algorithms** | **Malignancy:**  **Malignant tumor, n (%)** | **Topography: Lung, n (%)** | **Morphology: NSCLC, n (%)** | **Origin:**  **Primary, n (%)** |
| --- | --- | --- | --- | --- |
| **First version of the algorithm**  n=2003 patients from PR of Siena | Yes: 1992 (99.46%)  No: 2 (0.09%)  Maybe: 9 (0.45%) | Yes: 1754 (87.56%)  No: 180 (8.98%)  Maybe: 69 (3.44%) | Yes: 1836 (91.66%)  No: 93 (4.64%)  Maybe: 74 (3.69%) | Yes: 995 (49.67%)  No: 292 (14.57%)  Maybe: 716 (35.74%) |
| **Final version of the algorithm**  n=200 patients from PR of Tuscany | Yes: 200 (100%) | Yes: 190 (95%)  No: 1 (0.5%)  Maybe: 9 (4.5%) | Yes: 200 (100%) | Yes: 34 (17%)  No: 4 (2%)  Maybe: 162 (81%) |

PR: Pathology registry
